# False-negative finding in urodynamic study for the chief complaint. Does it interfere with the clinical outcomes for the treatment of SUI or OAB syndromes?

**DOI:** 10.1590/S1677-5538.IBJU.2020.0387

**Published:** 2020-12-20

**Authors:** Paulo Rodrigues, Flávio Hering, Eli Cielici, Marcio D´Império

**Affiliations:** 1 Hospital Beneficência Portuguesa de São Paulo Clínica de Urologia São PauloSP Brasil Clínica de Urologia, Hospital Beneficência Portuguesa de São Paulo -SP, Brasil; 2 Hospital Santa Helena de São Paulo Serviço de Urologia São Paulo Brasil Serviço de Urologia do Hospital Santa Helena de São Paulo -São Paulo, Brasil

**Keywords:** Urodynamics, Urinary Incontinence, Syndrome

## Abstract

**Purpose:**

False-negative urodynamic findings may mislead or prevent planned treatments due to unmatched findings with the clinical presentation. We hypothesized that the absence of urodynamic demonstration of SUI or OAB on urodynamics would interfere with clinical outcomes.

**Materials and Methods:**

Materials and Methods: We prospectively studied 124 women with (94) or without (30) demonstrable SUI after sling operations. Similarly, 64 women with OAB syndrome with (38) or without (26) demonstrable DO were also compared after treatment with anticholinergic agents. Patients were assessed with the UDI-6 and IIQ-7 questionnaires 3 and 6 months after treatment.

**Results:**

Only 76% of SUI patients demonstrated urine leakage during urodynamics. The UDI-6 score was higher in the demonstrable-SUI and demonstrable-DO groups, while the IIQ-7 score was comparable within the incontinence or urgency/frequency groups. Demonstrable and non-demonstrable SUI-operated patients showed similar outcomes. Patients with urgency syndromes with or without demonstrable DO had a similar rate of improvement with anticholinergic therapy.

**Conclusions:**

Women with clinical complaints of SUI objectively demonstrated on urodynamics presented the same subjective clinical outcome as those with SUI lacking objective demonstration when measured by the UDI-6 and IIQ-7 questionnaires. Similarly, patients with OAB syndrome with or without demonstrable DO had similar clinical improvement when treated with anticholinergics and measured using the same questionnaires.

## INTRODUCTION

For many urologists and most certainly for many referral centers and third-party payers, urodynamic studies are a necessary and prognostic tool to evaluate bladder behavior before any therapeutic approach is implemented (1) despite good evidence that it could be waived in index cases (2, 3).

Different routine laboratory tests, such as filling rates, catheter gauge, temperature of the infused fluid and patient-related physiological variations, may interfere with the detection rate of relevant information gathered during urodynamic study, confounding the reasons and first-hand expectations that prompted the assistant urologist to request the urodynamic investigation (4). Additionally, unexpected findings from ancillary exams may also affect prior planned treatment (5).

In that particular scenario, two situations may interfere:

1. Urodynamic findings do not correlate to the main complaint; and
2. Urodynamic findings prevent the diagnostic confirmation of the main complaint, resulting in a false-negative exam.

In many centers, legal problems or even third-party payers’ authorization of therapies or medications depend on objective documentation of the chief complaint (1). Moreover, the regional culture of medical practice may also determine the rate and acceptability of certain kinds of invasive investigations (6).

In our practice, urodynamic examination is an essential part of the medical workup to orient the intended treatment plan.

We hypothesized that the lack of demonstration of the main clinical complaint on the urodynamic study taken as a surrogate for the main diagnosis would impact the subjective clinical result measured by validated questionnaires in index SUI or OAB groups of patients. Subjective outcomes measured by validated questionnaires in surgically treated SUI (stress urinary incontinence) cases with or without demonstrated urinary leakage during the exam were studied. A second scenario comprising cases with clinically urgency-frequency syndrome (OAB) with or without demonstrated DO (detrusor overactivity) during urodynamic evaluation was also compared between groups after a 3-month period of anticholinergic treatment.

## MATERIAL AND METHODS

We prospectively studied 150 consecutive women treated with sub-urethral slings with clinical diagnosis of SUI as the main clinical complaint. Patients were studied and split into two groups according to the presence or absence of demonstrable urinary leakage on the first session of preoperative urodynamic evaluation as it is routinely done as part of our diagnostic workup and therapeutic plan for elective sling operation.

SUI patients were enrolled if they complained of involuntary urinary leakage under stress during daily life activities.

Similarly, 80 consecutive women with clinical complaints of urinary urgency-frequency syndrome (OAB) - frequency >10x/day and clinical complaints of urinary urgency without urinary leakage were also enrolled as the OAB arm. Baseline neurological conditions and urinary tract infection were ruled out. These patients were treated with an anticholinergic (solifenacin-10mg) as a first-line treatment. Before initiating any anticholinergic treatment, patients were routinely investigated with urodynamic study as part of our protocol as demanded by local health care providers. A 3-month period was empirically determined for clinical reevaluation with validated questionnaires.

Patients with a history of neurological disorder, urinary retention, bladder outlet obstruction or prior genitourinary surgeries were excluded from the study. To avoid enrolling potentially obstructed women, we restricted patients with Q_max_ <12mL/s, postvoid residual >100mL and P_det_Q_max_ >40cmH_2_O as exclusion criteria. No patients with grade III or IV prolapse were enrolled (7).

All women in both groups were asked to complete two short versions of urinary symptom questionnaires (UDI-6 and IIQ-7) before and at 3 and 6 months of follow-up in the surgical arm and before and at 3 months in the medication arm. The average IIQ-7 and UDI-6 total scores were multiplied by 33 to put them on a scale of 0 to 100.

Multichannel urodynamic studies (Aquarius XLT^®^, Laborie Medical Tech, Vermont, USA) were conducted. Urodynamic studies were performed according to the good urodynamic practices recommended by the ICS (8).

Patients were investigated in the sitting position after a free-flow rate was first conducted in private.

A double lumen 6Fr transurethral catheter was used to fill the bladder pressure at a filling rate of 50mL/min with a 37°C mixture of saline solution. At each 100mL interval, VLPP was conducted by asking the patient to perform the Valsalva maneuver (9). Women were asked to strain three attempts in a row, followed by three sets of coughing to detect any leakage of urine. If urine leakage could not be demonstrated, a final set of repeated maneuvers was performed with the patient standing or squatting before the exam ended.

Finally, patients were allowed to void in the sitting position, and a pressure-flow study was recorded when the patient manifested a strong desire to void.

DO was diagnosed if the increase on the graphic line was at least 5cmH2O in amplitude. This pressure cut-off was used because it represented the lowest detrusor pressure amplitude overtly detected with certainty as a true detrusor contraction. The highest detrusor pressure recorded during the filling phase was taken as the measured DO.

Cure of SUI was defined as no urine loss upon daily activities in a subjective evaluation.

Continuous variables were compared using t-tests, and dichotomous variables were compared using chi-square tests. Differences and correlations between paired values and occurrences of DO were analyzed by Wilcoxon´s signed rank test and Spearman rank correlation for p <0.05 in a 2-tailed analysis.

The authors consulted both Institutional Review Boards of the two involved hospitals (IRB-HSH-8742). All patients provided written informed consent for exams.

## RESULTS

The mean age of the studied population was 49 (±6.7; range 28 to 74), with an average parity of 2.1 (±0.4/range 0 to 4).

In the SUI arm, 124 of 150 (82%) patients were available for the study, and in the OAB arm, 64 of 80 (80%) completed the pre-and post-treatment questionnaires. Data were incomplete in 18 patients in the SUI group and 12 in the OAB group, while 8 patients in the SUI group and 4 patients in the OAB group did not return for appropriate follow-up.

Both arms of the study showed similar demographic data concerning age and parity (Table-1).

**Table 1:** Characteristics of 2 arm groups of women operated for SUI or treated with anticholinergic drugs.

	SUI demonstrated (n=94)	SUI not demonstrated (n=30)	p	DO demonstrated (n=38)	DO not demonstrated (n=26)	p
	SUI (+)	SUI (-)		OAB (+)	OAB (-)	
Age (mean ± SD)	53.1 (±4.4)	47.3 (±5.0)	N.S.	45.6 (±5.6)	41.83 (± 6.5)	N.S.
Parity(mean ± SD)	2.1 (±0.6)	1.8 (±0.3)	N.S.	2.0 (±0.6)	1.8 (± 0.3)	N.S.
Time of clinical perception for SUI (year)(mean ± SD)	5.5 (±2)	3.4 (±0.8)	N.S.	*	*	N.S.
Time of clinical perception for Urgency (months) (mean ± SD)	Not available	Not available	N.S.	10.5 (±9.8)	11.1 (± 6.2)	N.S.
Urge-incontinence symptoms	Not available	Not available	N.S.	39.4% (15)	30.7% (8)	N.S.
Urethral hypermotility (n,%)	81 (86%)	27 (90%)	N.S.	29 (76%)	22 (84%)	N.S.
Urinary leakage detected during office examination	80 (88.2%)	5 (16.6%)	0.001	6 (15.5%)	7 (28%)	0.04

**n.s.** - not significant;

* - irrelevant or not applicable

Out of 124 cases in the SUI arm, 68% (85/124) had urinary leakage demonstrated at the office examination by cough, straining and jumping maneuvers.

Seventy-six percent (76%) of the patients (94/124) leaked urine during the urodynamic study.

Eighty-seven percent (108/124) of the patients who had urinary leakage leaked urine on either of the two attempts to demonstrate SUI, but only 47.2% (51/108) of the leaking cases leaked urine at the office and during urodynamic exam.

Sixteen cases (12.9%) did not leak urine during the office maneuvers or at the urodynamic study (p <0.001).

Demonstrable SUI patients presented DO during the exam more frequently than patients with no demonstrable SUI (p=0.03). In a similar fashion, demonstrable SUI patients had higher UDI-6 and IIQ-7 scores than those not leaking at the study (Table-2).

**Table 2:** Urodynamic findings of 2 arm groups of women submitted to urodynamic evaluation treated for SUI or OAB.

	SUI demonstrated (n=94)	SUI not demonstrated (n=30)	P	DO demonstrated (n=38)	DO not demonstrated (n=26)	p
Q_max_	18.5 (±5.3)	16 (±8.1)	n.s	24.8 (±8.8)	26.1 (± 9.5)	n.s.
Residual after free flow rate (mL)	12.1 (±7)	7.8 (±5)	0.05	18.9 (±4)	3 (± 3)	0.01
P_det_Q_max_ (cmH_2_O)	23.9 (±17)	20.8 (±9)	n.s.	25.9 (±7)	21.2 (± 11)	0.04
Voided volume (mL)	276 (±75)	256 (±31)	n.s.	189 (±22)	215 (± 35)	0.03
Urodynamically Detected detrusor overactive	18.1% (17)	13.3% (4)	0.03	100% (38)	0% (26)	0.04
Amplitude of detrusor overactive contraction –(cmH_2_O)	16 (±7)	21.7 (±7.6)	0.02	17.7 (±12.3)	Not available	*
Urinary leakage detected at urodynamic exam	100% (94)	0% (0)	*	21% (8)	7.7% (2)	0.02
VLPP	58	Not available	*	78 (±19)	108 (± 14)	0.02
Maximum generated VLPP (cmH_2_O)	103 (±23)	147 (±87)	n.s.	89 (±34)	107 (± 35)	n.s.
Maximum bladder capacity (mL)	376 (±56)	424 (±87)	0.07	189 (±56)	221 (± 78)	0.03
Pre-op UDI-6 (urinary symptoms score)	52 (±15)	45 (±17)	0.04	64 (±22)	53 (± 14)	0.04
Post-op UDI-6 (urinary symptoms score)	14 (±12)	13 (±11)	n.s.	39 (±17)	33 (± 9)	n.s.
Pre-op IIQ-7 (quality of life)	62 (±23)	51 (±20)	0.04	57 (±21)	46 (± 17)	0.03
Post-op IIQ-7 (quality of life)	17 (±16)	15 (±13)	n.s.	47 (±7)	36 (± 9)	0.04

n.s. - not significant;

* - irrelevant or not applicable

The final subjective outcomes obtained from patients who underwent surgery for SUI measured by UDI-6 and IIQ-7 were equivalent for the groups who leaked or did not leak at the urodynamic study (p=0.8).

In the OAB patient group, residual volume after Q_max_, P_det_Q_max_, voided volume and urine leaking during urodynamics were significantly different for those who did or did not demonstrate DO during the urodynamic study (Table-2).

Urinary leakage by the Valsalva maneuver was more prevalent in the demonstrable-DO group than in those who did not present DO during the exam (p=0.02). This finding may be related to the fact that demonstrable DO cases in urodynamic studies presented lower VLPP when compared to those without demonstrable DO.

Although the UDI-6 and IIQ-7 scores were higher for patients with demonstrable DO in comparison to patients lacking demonstrable DO, the subjective improvement observed after medical treatment for OAB in both groups was comparable and not statistically significant between the two groups. The same trend was observed when the clinical complaints were measured by the IIQ-7 questionnaire.

A final comparison between the SUI and OAB groups revealed that patients treated for SUI subjectively improved much more than the patients treated for OAB syndrome when measured using UDI-6 and IIQ7 (p <0.001).

## DISCUSSION

Urodynamics is a preliminary attempt to reproduce the symptoms or signs of urinary complaints in a non-physiological setting.

As expected, urodynamics should reveal bladder behavior as an extension of the chief clinical complaint in a perfect match, helping the assistant doctor choose the best management course and promote a realistic conversation about outcomes for the patient’s treatment.

However, urodynamic exams may not reproduce the leading clinical complaint, or the habitual voiding pattern does not mirror daily bladder function (4), and its use has been criticized because of its low sensitivity rate.

False-negative results are not well understood, but patient efforts to prevent urinary leakage and the artificial setting of the exam may account for these variations (9, 10).

Regarding SUI and frequency/urgency syndromes, if urodynamic investigation does not confirm the clinical suspicion, it may alter the intended treatment plan, posing a quandary to the assistant doctor who may feel misguided by the achieved urodynamic result despite the vivid clinical complaint. Failure to demonstrate urinary leakage in patients with clinical SUI may be as high as 50% (11).

A Dutch survey demonstrated that the detection of DO on urodynamics would modify the therapeutic plan for 87% of the attendant gynecologists and for 80% of interviewed urologists (5), as lower subjective satisfactory rates were observed for women submitted to sub-urethral slings for preoperative DO (12).

In some parts of the World, legal and third-party payer policies may impose objective documentation of urinary leakage or DO to authorize invasive or medical treatments such as botulinum toxin injection.

We recently confirmed that DO was demonstrated in only 44% of the patients at the first filling cystometry, suggesting that repetition of cystometry in the same session might be mandatory (13).

Our present data revealed that patients without demonstrated DO presented larger bladder capacity, a lower rate of SUI and higher VLPP but with no significance on the subjective improvement measured by UDI-6 or IIQ-7 after anticholinergic treatment was initiated (p >0.05), which is in parallel to Balachandran, who demonstrated the lack of correlation between demonstrable DO and the Patient Global Impression of Improvement (PGI-I) questionnaire for those treated with mirabegron (14). Surprisingly, patients complaining of urinary urgency with demonstrable DO were not worse than those who did not have it demonstrated at the exam.

Our sling-operated patients also did not show differences in the rate or amplitude of DO, maximum generated VLPP or pre-or postoperative clinical scores measured by UDI-6 or IIQ-7 (p >0.05). SUI patients who leaked urodynamically were not more satisfied than those who did not leak.

Our higher rate of demonstrable urinary leakage during the exam in SUI cases (76%-94 in 124 cases) may be the result of our unrelenting effort to demonstrate it during the course of the exam or the uniqueness of the studied population, as 78.1% also leaked at the physical exam.

Caruso et al. correlated urodynamic studies and clinical findings using the same UDI-6 as we did and reported that 54.4% of their patients did not leak during urodynamic maneuvers, while less than 1% of their population leaked during the exam if the patient denied SUI (15). They concluded that demonstration of SUI on urodynamics was significantly related to the clinical complaint of SUI on history, although there was still a high failure of objective demonstration. Those same authors observed only a 30% rate of concordance between clinical and urodynamic confirmation for DO (15) and a 21% rate of discordance on DO in cases with clinical urgency/frequency complaints. In our population, 68% of our patients with clinical complaints of urgency had confirmatory DO on urodynamic investigation. The reasons for this are not clear, but it may be related to our assumed lower cut-off for DO diagnosis.

In a large randomized clinical trial with 1233 women who underwent sling operations, 11.7% of the patients did not show urine leakage during preoperative urodynamic exams (16).

We acknowledge that visual or clinical demonstration of urine leakage during urodynamic studies may not be reproducible because protocols vary along the diameter of the catheter (17) and bladder volume in which the maneuvers are elicited (9).

The exact significance of not having SUI demonstrated at the preoperative evaluation demands more studies, as one author showed better clinical results in women demonstrating urinary leakage versus those not showing it (17), which contradicts our observation.

Both conditions (OAB and SUI) demonstrated a clinical response to their respective treatments, but patients with SUI showed a more remarkable improvement when compared to those in the OAB arm.

In addition, OAB seems to be more bothersome and a recalcitrant condition reflected by the higher pre-treatment score and less impressive outcome when compared to the SUI-treated population.

Our permissive use of urodynamic studies and third-party requirements to authorize treatments before any intervention allowed a unique opportunity to compare those with urodynamic confirmation of the chief complaint with those who did not show it. Taking advantage of this position, we demonstrated that unmatched clinical and urodynamic findings have little or no impact on subjective clinical response measured by validated questionnaires.

Our study has limitations because we assumed that clinical complaints of SUI and frequency-urgency syndrome were surrogates for the urodynamic diagnosis of those conditions, and repetition of the exam in the same session might have enhanced the specificity of the urodynamic findings.

## CONCLUSIONS

In conclusion, women with clinical complaints of SUI or OAB syndrome lacking significant genital prolapse or putative urodynamic obstruction showed the same rate of clinical improvement measured by two different validated questionnaires although their clinical complaints may or may not be represented on the urodynamic lines.

## References

[1] 1. Zimmern P, Litman H, Nager C, Sirls L, Kraus SR, Kenton K, et al. Pre-operative urodynamics in women with stress urinary incontinence increases physician confidence, but does not improve outcomes. Neurourol Urodyn. 2014; 33:302-6. 10.1002/nau.22398 PMC3830633 23553613

[2] 2. Lloyd JC, Dielubanza E, Goldman HB. Trends in urodynamic testing prior to midurethral sling placement-What was the value of the VALUE trial? Neurourol Urodyn. 2018; 37:1046-52. 10.1002/nau.23398 28877362

[3] 3. Nager CW, Brubaker L, Litman HJ, Zyczynski HM, Varner RE, Amundsen C, et al. A randomized trial of urodynamic testing before stress-incontinence surgery. N Engl J Med. 2012;366:1987-97. 10.1056/NEJMoa1113595 PMC3386296 22551104

[4] 4. van Venrooij, G. E., & Boon, T. A. (1994). Extensive urodynamic investigation: interaction among diuresis, detrusor instability, urethral relaxation, incontinence and complaints in women with a history of urge incontinence. The Journal of urology, 152 (5 Pt 1), 1535–8. 10.1016/s0022-5347(17)32464-3 7933194

[5] 5. van Leijsen SA, Kluivers KB, Mol BW, Vierhout ME, Heesakkers JP. The value of preoperative urodynamics according to gynecologists and urologists with special interest in stress urinary incontinence. Int Urogynecol J. 2012; 23:423-8. 10.1007/s00192-011-1565-2 PMC3305873 21927939

[6] 6. Brown P, Lyson M, Jenkins T. From diagnosis to social diagnosis. Soc Sci Med. 2011; 73:939-43. 10.1016/j.socscimed.2011.05.031 21705128

[7] 7. Pfisterer MH, Griffiths DJ, Rosenberg L, Schaefer W, Resnick NM. Parameters of bladder function in pre-, peri-, and postmenopausal continent women without detrusor overactivity. Neurourol Urodyn. 2007; 26:356-61. 10.1002/nau.20381 17285577

[8] 8. Drake MJ, Doumouchtsis SK, Hashim H, Gammie A. Fundamentals of urodynamic practice, based on International Continence Society good urodynamic practices recommendations. Neurourol Urodyn. 2018; 37:S50-S60. 10.1002/nau.23773 30614058

[9] 9. Rodrigues P, Afonso Y, Hering FO, Campagnari JC, Azoubel A. Valsalva leak point pressure to determine internal sphincter deficiency in stress urinary incontinence. Urol Int. 2006; 76:154-8. 10.1159/000090880 16493218

[10] 10. Liversidge K, Guzmán Rojas R, Kamisan Atan I, Dietz HP. Negative urodynamic testing in women with stress incontinence. Aust N Z J Obstet Gynaecol. 2015; 55:76-80. 10.1111/ajo.12290 25494576

[11] 11. Caruso DJ, Kanagarajah P, Cohen BL, Ayyathurai R, Gomez C, Gousse AE. What is the predictive value of urodynamics to reproduce clinical findings of urinary frequency, urge urinary incontinence, and/or stress urinary incontinence? Int Urogynecol J. 2010; 21:1205-9. 10.1007/s00192-010-1180-7 20559620

[12] 12. Kulseng-Hanssen S, Husby H, Schiotz HA. The tension free vaginal tape operation for women with mixed incontinence: Do preoperative variables predict the outcome? Neurourol Urodyn. 2007; 26:115-21. Erratum in: Neurourol Urodyn. 2008; 27:360. 10.1002/nau.20597 18404618

[13] 13. Rodrigues P, Hering F, Cieli E, D’Imperio M, Campagnari JC. Can We State Stable Bladder? How Many Repetitions Should We Do for an Appropriate Demonstration of Involuntary Detrusor Contraction? Urol Int. 2015; 95:86-91. 10.1159/000370163 25661681

[14] 14. Basu M, Balachandran A, Duckett J. Is pretreatment cystometry important in predicting response to mirabegron in women with overactive bladder symptoms? Int Urogynecol J. 2016; 27:427-31. 10.1007/s00192-015-2809-3 26282091

[15] 15. Caruso DJ, Kanagarajah P, Cohen BL, Ayyathurai R, Gomez C, Gousse AE. What is the predictive value of urodynamics to reproduce clinical findings of urinary frequency, urge urinary incontinence, and/or stress urinary incontinence? Int Urogynecol J. 2010; 21:1205-9. 10.1007/s00192-010-1180-7 20559620

[16] 16. Lemack GE, Litman HJ, Nager C, Brubaker L, Lowder J, Norton P, et al. Preoperative clinical, demographic, and urodynamic measures associated with failure to demonstrate urodynamic stress incontinence in women enrolled in two randomized clinical trials of surgery for stress urinary incontinence. Int Urogynecol J. 2013; 24:269-74. 10.1007/s00192-012-1821-0 PMC4169424 22669421

[17] 17. Türker P, Kilic G, Tarcan T. The presence of transurethral cystometry catheter and type of stress test affect the measurement of abdominal leak point pressure (ALPP) in women with stress urinary incontinence (SUI). Neurourol Urodyn. 2010; 29:536-9. 10.1002/nau.20801 19693953

